# Crystal structure of 2-methyl-1,2,3,4-tetra­hydro­iso­quinoline trihydrate

**DOI:** 10.1107/S2056989020000730

**Published:** 2020-02-06

**Authors:** Felix Langenohl, Felix Otte, Carsten Strohmann

**Affiliations:** a Technical University Dortmund, Inorganic Chemistry, Otto-Hahn-Strasse 6, D-44227, Dortmund, Germany

**Keywords:** crystal structure, 2-methyl-1,2,3,4-tetra­hydro­iso­quinoline, TIQ, heterocyclic amine, secondary amine, hydrogen bonding, crystal water

## Abstract

The crystal structure of the title compound, C_10_H_13_N·3H_2_O, a heterocyclic amine, was determined in the presence of water. The compound co-crystallizes with three water mol­ecules in the asymmetric unit, which leads to the formation of hydrogen bonding in the crystal.

## Abstract 2

The crystal structure of the title heterocyclic amine was determined in the presence of water. The compound co-crystallizes with three water mol­ecules in the asymmetric unit, which leads to the formation of hydrogen bonding in the crystal.

## Chemical context   

Tetra­hydro­iso­quinolines are heterocyclic secondary amines that can be found in animal and human brains (Rommelspacher & Susilo, 1985 ▸). Many compounds of this class and their derivatives are bioactive and show promising pharmacological potential, for example as neuroprotectants or anti­tumor anti­biotics (Scott & Williams, 2002 ▸; Antkiewicz-Michaluk *et al.*, 2014 ▸). Studies show that some of these endogenous compounds function as Parkinsonism-inducing agents, while others can prevent Parkinsonism and are therefore promising candidates for treatment of Parkinson’s disease (Kotake *et al.*, 1995 ▸; Lorenc-Koci *et al.*, 1999 ▸, 2008 ▸; McNaught *et al.*, 1998 ▸; Storch *et al.*, 2002 ▸). Their structures are therefore analysed to gain a better understanding of their function and possible chemical and pharmaceutical properties. In this case, we report the crystal structure of 2-methyl-1,2,3,4,-tetra­hydro­iso­quinoline, which co-crystallizes with water.
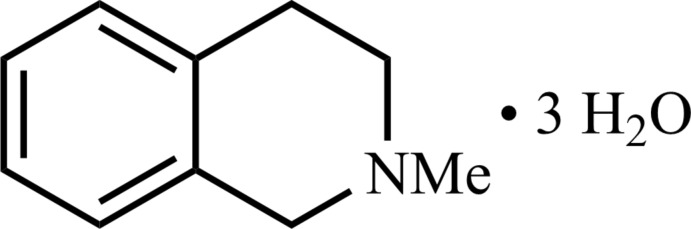


## Structural commentary   

**Figure 1:**
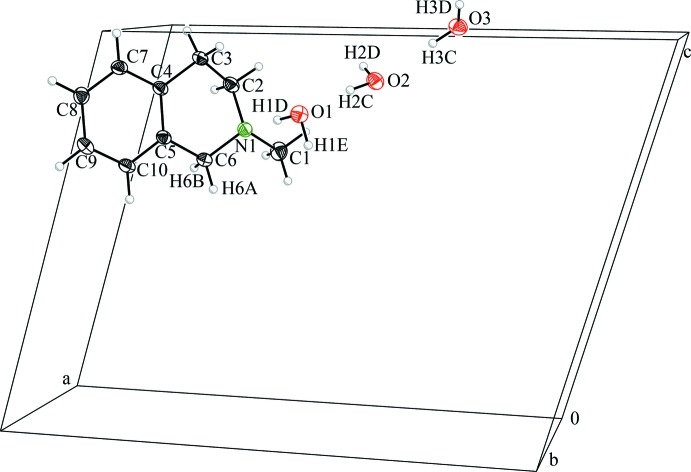
Asymmetric unit and mol­ecular structure in the crystal of the title compound with the unit-cell boundaries and atom labelling. Displacement ellipsoids are drawn at the 50% probability level.

The heterocyclic amine, itself an oil at room temperature, crystallizes in the presence of water at 243 K, and crystals are stable up to *ca* 273 K when they melt. The asymmetric unit of the mol­ecular structure, in space group *P*2_1_/*c*, is illustrated in Fig. 1 ▸. In addition to the heterocyclic amine, the asymmetric unit contains three water mol­ecules, which make up 27 mass % of the crystal. For poorly crystallizing organic compounds containing hydrogen-bond acceptors with weak polar inter­actions (such as the title compound), crystallization in the presence of water and therefore the formation of hydrate compounds seems to be an alternative strategy for crystal formation and/or purification. This holds true especially when the formation of ions, *e.g*. hydro­chlorides, is not desired to avoid structural changes caused by derivatization of the compounds.

The amine exhibits typical bond lengths and angles in the expected ranges (Allen *et al.*, 1987 ▸). The compound contains two different ring systems. The aromatic ring (C4/C5/C7–C10) is planar as expected, while the non-aromatic ring (N1/C2-C6) has a half-boat conformation and can be described with the Cremer–Pople parameters with a total puckering amplitude of *Q*_T_ = 0.5067 (11) Å, an azimuthal angle (*θ*) of 133.22 (12)° and a zenithal angle (*Φ*) of 208.82 (18)°. The structure is comparable with those of other tetra­hydro­isochinoline derivatives such as 2-(2-chloro­acet­yl)-6,7-dimeth­oxy-1,2,3,4-tetra­hydro­iso­quinoline (Ling *et al.*, 2006 ▸) or 5-(6,7-dimeth­oxy-1,2,3,4-tetra­hydro­isoquinolin-2-yl)-4-phenyl-1,2,5-oxa­diazole *N*-oxide (Xu *et al.*, 2006 ▸), that also show a half-boat conformation of the non-planar ring. The nitro­gen atom displays a tetra­hedral environment, which indicates an *sp* ^3^ hybridization, as is to be expected for a tertiary amine. This is similar to the tetra­hydro­iso­quinoline published by Xu *et al.*, but in comparison the mentioned structure from Ling *et al.* shows a trigonal planar *sp* ^2^-hybridized nitro­gen atom. Some selected bond lengths and angles are listed in Table 1 ▸.

## Supra­molecular features   

As a result of the high amount of crystal water, an extensive supra­molecular hydrogen-bonding network is formed. Geometrical details of the hydrogen bonding are listed in Table 2 ▸.

**Figure 2:**
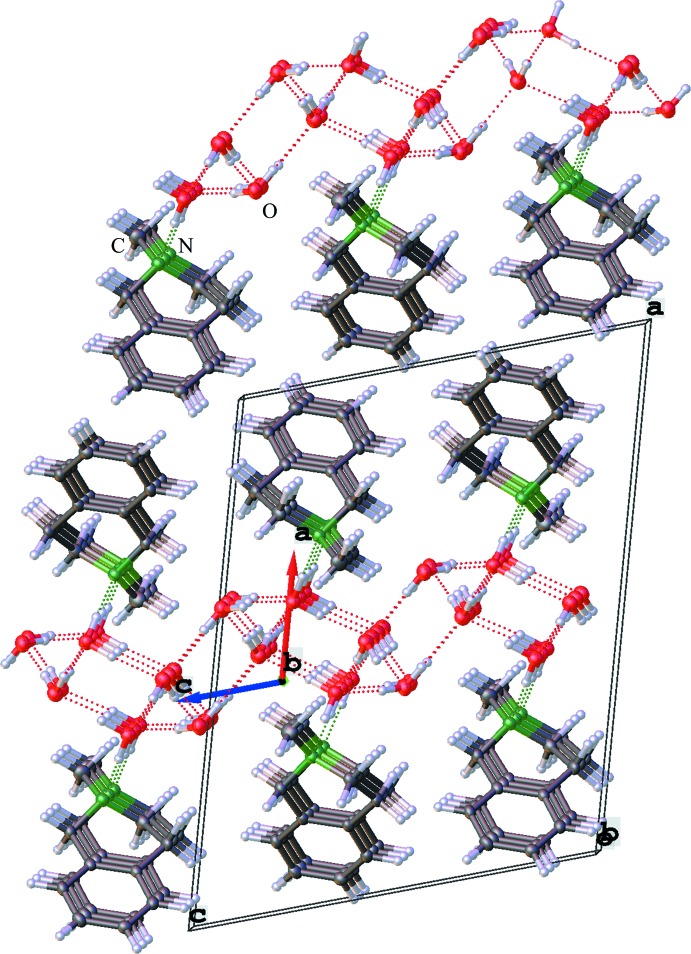
View along the *b* axis through the crystal packing shows the hydrogen-bonding network, the parallel stacked organic mol­ecules and reveals the alternating hydro­phobic and hydro­philic packing phases.

The crystal water forms a matrix in the *bc* plane, to which the amines are bound with the help of another set of hydrogen bonds. A section of the supra­molecular hydrogen bonding and crystal packing along the *b*-axis direction is shown in Fig. 2 ▸. In this view the water forms a channel along the *c* axis, and the bridging of the organic mol­ecules by the nitro­gen atoms is clearly visible. The organic mol­ecules are stacked in parallel along the *b* axis with a distance of 5.9209 (6) Å. The iso­quinolines on the other side of the infinite water channel are invertedly aligned along the *c* axis. This also results in the formation of alternating hydro­philic and hydro­phobic phases of the hydrogen-bonded water framework and organic phases of the heterocyclic amines along the *a* axis.

**Figure 3:**
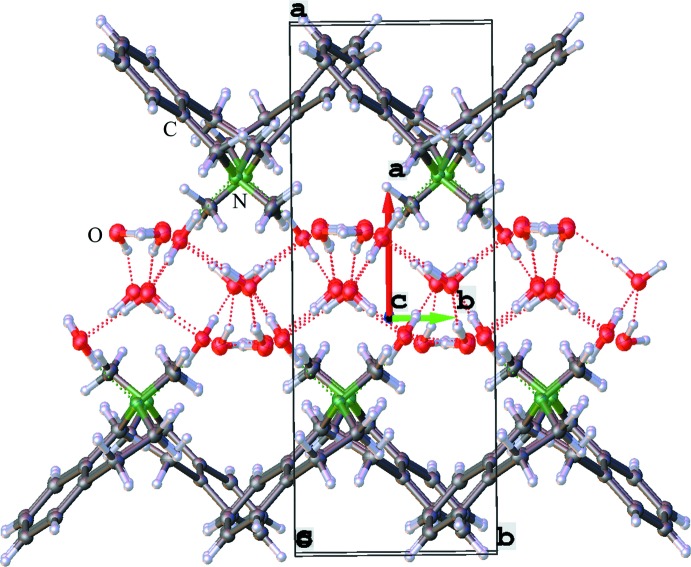
View along the *c* axis through the crystal packing showing the other side of the hydrogen-bonding network and the different arrangement of the organic mol­ecules.

An alternative view of the crystal packing along the *c* axis shows that the heterocyclic amines are alternately connected to the hydrogen-bonding system along the axis, which leads in the formation of syndiotactic polymer chains in this dimension (see Fig. 3 ▸).

**Figure 4:**
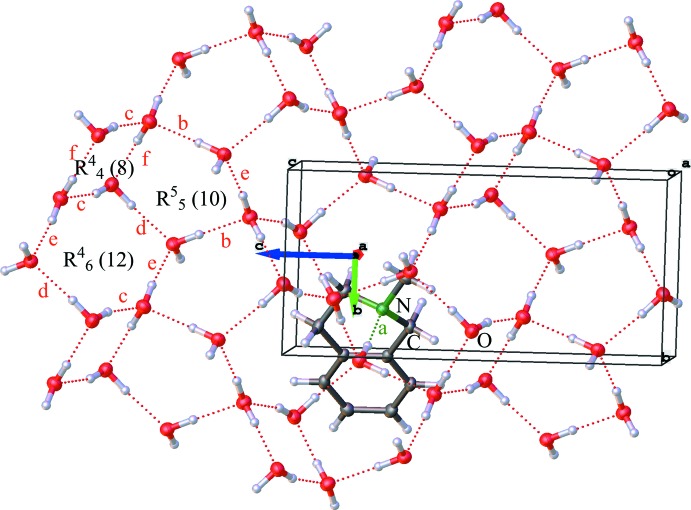
View along the *a* axis through the crystal packing shows the hydrogen-bonding network. For a better view, only one amine mol­ecule is shown, to highlight the supra­molecular water network in the *bc* plane. The various hydrogen bonds are labelled as examples for a four-, five- and sex-membered ring (red *b*–*f*), as well as an amine hydrogen bond (green *a*).

An analysis of the hydrogen-bonding network formed by the water mol­ecules is illustrated in Fig. 4 ▸. Here the view along the *a* axis shows the formed water plane along the *b* and *c* axes with different ring systems (only counting the oxygen atoms) and the graph-set motifs of the hydrogen-bonding network. The infinite hydrogen-bonded network is formed along the *c* axis by chains of connected five-membered [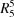 (10)] rings (connected *via* hydrogen bond *b*) followed by chains of alternating four- and six-membered [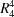 (8)] and [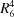 (12)] rings (connected *via* hydrogen bond *c*) that are orientated along the *b* axis.

**Figure 5:**
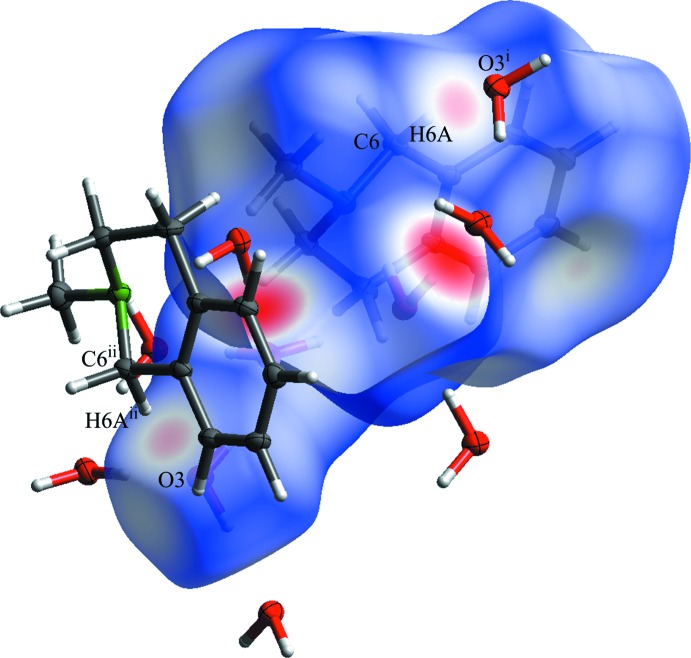
Hirshfeld-surface analysis (*CrystalExplorer17*; Turner *et al.*, 2017 ▸) of the compound displays close contacts in the crystal. The weak hydrogen bond between oxygen atom O3 and the H6*A* hydrogen atom is labelled. Symmetry codes: (i) 1 – x, 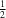 + *y*, 3/2 – z; (ii) 1 – x, 1 – y, 2 – z.

For the third oxygen atom (O3), the ideal tetra­hedral environment (Bernal & Fowler, 1933 ▸) is achieved by formation of a weak hydrogen bond to the H6*A* hydrogen atom of the *alpha* carbon atom (C6), which is indicated by the short C6⋯O3 distance [3.4531 (3) Å]. This can be highlighted by an Hirshfeld surface analysis, shown in Fig. 5 ▸. The short distance alone is not a clear evidence for a weak hydrogen bond, however the linear angle C6—H6*A*⋯O3 of 168.8 (2)° (without cone-correction; Kroon & Kanters, 1974 ▸) strongly supports this assumption. For an overview of the definition and characteristics of weak hydrogen bonding, see, for example: Desiraju & Steiner (1999 ▸).

## Database survey   

A survey of the Cambridge Crystallographic Database (CSD, Version 5.40, September 2019; Groom *et al.*, 2016 ▸) shows about 1000 results for structures where the investigated amine is a substructure of a more complex structure. The before-mentioned compounds C_13_H_16_ClNO_3_ (Ling *et al.*, 2006 ▸) and C_20_H_21_N_3_O_4_ (Xu *et al.*, 2006 ▸) are two examples of this. Moreover, some others are C_19_H_17_NO_5_ (Aree *et al.*, 2003 ▸), C_24_H_23_NO_2_ (Philippe *et al.*, 2000 ▸), C_15_H_17_NO_3_ (Li *et al.*, 2011 ▸) and C_22_H_23_NO_6_ (Roques *et al.*, 1978 ▸). Another example for a reported crystal structure is C_24_H_25_NO_3_·2CH_3_OH, which is used as a PET radiotracer and has been tested in clinical evaluation for early diagnosis of Alzheimer’s disease (Altomare *et al.*, 2014 ▸). All derivates found during the survey have in common that they have more complex structures and are often *O*-functionalized compared to the title compound. Some small reported analogues of this compound are metallated derivatives with lithium and potassium, which were published in a study about stabilization of different amine anions by our group (Unkelbach *et al.*, 2012 ▸).

## Synthesis and crystallization   

1,2,3,4-Tetra­hydro­iso­quinoline (10 mL, 79.66 mmol) was dissolved in 30 mL of formic acid (99%). After adding formaldehyde (30 mL, 37% in water) the solution was stirred under reflux for 6 h and stirred at room temperature for an additional 12 h. Subsequently KOH was added to adjust to pH 13. In the next step, the two-phase system was extracted with diethyl ether (3 x 50 mL). The combined organic phases were dried with MgSO_4_. After removing the solvent the raw product was distilled (333 K, 0.25 mbar) and the pure amine could be obtained as a colourless oil (94% yield).

The title compound crystallizes in the presence of water, by adding some drops of water to a solution of the amine in Et_2_O, mixing the two phases and then separating again to obtain a moist organic phase. Storage of the organic phase at 243 K results in crystallization of the title compound in colourless needles, which are stable up to 273 K before they start melting. The crystals were therefore selected for measurement with help of a X-Temp 2 low-temperature stage (Heine & Stalke, 1992 ▸; Stalke, 1998 ▸).

The pure amine is known from the literature and the measured analytical NMR and MS data correspond to the reported data (Locher & Peerzada, 1999 ▸).

^1^ *H* NMR (CDCl_3_, 400 MHz): δ (ppm) 2.48 (3H, *s*, NC*H*_3_), 2.72 (2H, *t*, NCH_2_C*H*_2_), 2.95 (2H, *t*, NC*H*_2_CH_2_), 3.61 (2H, *s*, NC*H*_2_C_ar_, 7.02–7.04 (1H, *m*, C*H*_ar_), 7.11–7.15 (3H, *m*, C*H*_ar_).

^13^ *C* NMR (CDCl_3_, 100 MHz): δ (ppm) 29.1 (1C, C_ar_ *C*H_2_CH_2_), 46.0 (1C, N*C*H_3_), 52.8 (N*C*H_2_CH_2_), 57.9 (C_ar_ *C*H_2_N), 125.6 (1C, *C*_ar_), 126.4 (1C, *C*_ar_), 128.6 (1C, *C*_ar_), 133.7 (1C, *C*_ar_), 134.5 (1C, *C*_ar_).

GC/MS (EI) *m*/*z* (intensity %): 146 (100) [M–H]^+^ 131 (9) [M–CH3–H]^+^, 104 (51).

Elemental analysis calculated (%) for C_10_H_13_Ni: C 81.6, H 8.9, N 9.5; found: C 81.2, H 9.0, N 9.6.

Because of the low stability of the crystals of the trihydrate, no further analysis of the trihydrate was carried out, except for NMR spectroscopy of the crystals, which reveals a broadened water signal in the ^1^ *H* NMR spectrum, which overlaps with other signals in *d*-aceto­nitrile.

## Refinement   

Crystal data, data collection and structure refinement details are summarized in Table 3 ▸. The C-bound hydrogen atoms of the amine, except the protons H6*A* and H6*B*, were included in calculated positions with C—H = 0.95 Å, *U*_iso_(H) = 1.2*U*_eq_(C) for aromatic hydrogen atoms, C—H = 0.99 Å, *U*_iso_(H) = 1.2*U*_eq_(C) for CH_2_ hydrogen atoms and with C—H = 0.98 Å, *U*_iso_(H) = 1.5 *U*_eq_(C) for methyl hydrogen atoms. All other protons were located in the difference-Fourier maps and refined freely.

## Supplementary Material

Crystal structure: contains datablock(s) I. DOI: 10.1107/S2056989020000730/zl2766sup1.cif

Structure factors: contains datablock(s) I. DOI: 10.1107/S2056989020000730/zl2766Isup2.hkl

Supporting information file. DOI: 10.1107/S2056989020000730/zl2766Isup3.cml

CCDC reference: 1979129

Additional supporting information:  crystallographic information; 3D view; checkCIF report

## Figures and Tables

**Table 1:** Selected geometric parameters (Å, °)

N1—C1	1.4678 (14)	N1—C6	1.4671 (13)
N1—C2	1.4708 (14)	C4—C5	1.3977 (13)
			
C1—N1—C2	110.91 (9)	N1—C2—C3	109.72 (9)
C6—N1—C1	109.43 (8)	N1—C6—C5	112.77 (8)
C6—N1—C2	110.12 (8)	C4—C3—C2	112.45 (9)

**Table 2:** Hydrogen-bond geometry (Å, °)

*D*—H⋯*A*	*D*—H	H⋯*A*	*D*⋯*A*	*D*—H⋯*A*
O1—H1*D*⋯N1	0.94 (2)	1.81 (2)	2.7394 (12)	170.7 (18)
O1—H1*E*⋯O2^i^	0.95 (2)	1.81 (2)	2.7635 (12)	176 (2)
O3—H3*C*⋯O2	0.86 (2)	1.96 (2)	2.8070 (13)	165.8 (19)
O3—H3*D*⋯O1^ii^	0.91 (2)	1.84 (2)	2.7461 (12)	175.1 (19)
O2—H2*C*⋯O1	0.92 (2)	1.84 (2)	2.7538 (12)	174.9 (19)
O2—H2*D*⋯O3^iii^	0.88 (2)	1.88 (2)	2.7512 (13)	173 (2)
C6—H6*A*⋯O3^i^	0.98 (2)	2.49 (2)	3.4531 (3)	168.8 (2)

Symmetry codes: (i) 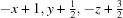; (ii) 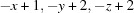; (iii) 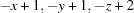.

**Table 3:** Experimental details

Crystal data	
Chemical formula	C_10_H_13_N·3H_2_O
*M*_r_	201.26
Crystal system, space group	Monoclinic, *P*2_1_/*c*
Temperature (K)	100
*a*, *b*, *c* (Å)	16.1791 (19), 5.9209 (6), 12.5007 (14)
β (°)	106.093 (5)
*V* (Å^3^)	1150.6 (2)
*Z*	4
Radiation type	Mo *K*α
μ (mm^−1^)	0.09
Crystal size (mm)	0.51 × 0.09 × 0.05

Data collection	
Diffractometer	Bruker D8 Venture
Absorption correction	Multi-scan (*SADABS*; Bruker, 2016 ▸)
*T*_min_, *T*_max_	0.655, 0.746
No. of measured, independent and observed [*I* > 2σ(*I*)] reflections	13488, 3350, 2708
*R*_int_	0.042
(sin θ/λ)_max_ (Å^−1^)	0.703

Refinement	
*R*[*F* ^2^ > 2σ(*F* ^2^)], *wR*(*F* ^2^), *S*	0.041, 0.109, 1.03
No. of reflections	3350
No. of parameters	160
H-atom treatment	H atoms treated by a mixture of independent and constrained refinement
Δρ_max_, Δρ_min_ (e Å^−3^)	0.35, −0.20

Computer programs: *APEX3* (Bruker, 2018 ▸), *SAINT* (Bruker, 2016 ▸), *SHELXT* (Sheldrick, 2015*a* ▸), *SHELXL* (Sheldrick, 2015*b* ▸), *OLEX2* (Dolomanov *et al.*, 2009 ▸), *PLATON* (Spek, 2020 ▸), *publCIF* (Westrip, 2010 ▸) and *Mercury* (Macrae *et al.*, 2008 ▸).

## supplementary crystallographic information

### Crystal data

**Table:**

C_10_H_13_N·3H_2_O	*F*(000) = 440
*M**_r_* = 201.26	*D*_x_ = 1.162 Mg m^−^^3^
Monoclinic, *P*2_1_/*c*	Mo *K*α radiation, λ = 0.71073 Å
*a* = 16.1791 (19) Å	Cell parameters from 5564 reflections
*b* = 5.9209 (6) Å	θ = 2.6–30.5°
*c* = 12.5007 (14) Å	µ = 0.09 mm^−^^1^
β = 106.093 (5)°	*T* = 100 K
*V* = 1150.6 (2) Å^3^	Needle, colourless
*Z* = 4	0.51 × 0.09 × 0.05 mm

### Data collection

**Table:**

Bruker D8 Venture diffractometer	3350 independent reflections
Radiation source: microfocus sealed X-ray tube, Incoatec Iµs	2708 reflections with *I* > 2σ(*I*)
HELIOS mirror optics monochromator	*R*_int_ = 0.042
Detector resolution: 10.4167 pixels mm^-1^	θ_max_ = 30.0°, θ_min_ = 2.6°
ω and φ scans	*h* = −22→22
Absorption correction: multi-scan (SADABS; Bruker, 2016)	*k* = −8→8
*T*_min_ = 0.655, *T*_max_ = 0.746	*l* = −17→17
13488 measured reflections	

### Refinement

**Table:**

Refinement on *F*^2^	Primary atom site location: dual
Least-squares matrix: full	Secondary atom site location: structure-invariant direct methods
*R*[*F*^2^ > 2σ(*F*^2^)] = 0.041	Hydrogen site location: mixed
*wR*(*F*^2^) = 0.109	H atoms treated by a mixture of independent and constrained refinement
*S* = 1.03	*w* = 1/[σ^2^(*F*_o_^2^) + (0.045*P*)^2^ + 0.3326*P*] where *P* = (*F*_o_^2^ + 2*F*_c_^2^)/3
3350 reflections	(Δ/σ)_max_ = 0.001
160 parameters	Δρ_max_ = 0.35 e Å^−^^3^
0 restraints	Δρ_min_ = −0.20 e Å^−^^3^

### Special details

**Table:**

Geometry. All esds (except the esd in the dihedral angle between two l.s. planes) are estimated using the full covariance matrix. The cell esds are taken into account individually in the estimation of esds in distances, angles and torsion angles; correlations between esds in cell parameters are only used when they are defined by crystal symmetry. An approximate (isotropic) treatment of cell esds is used for estimating esds involving l.s. planes.

### Fractional atomic coordinates and isotropic or equivalent isotropic displacement parameters (Å^2^)

**Table:**

	*x*	*y*	*z*	*U*_iso_*/*U*_eq_	
O1	0.59406 (5)	1.05257 (14)	0.81663 (7)	0.02252 (18)	
H1D	0.6378 (12)	0.971 (3)	0.7983 (16)	0.056 (5)*	
H1E	0.5579 (14)	1.109 (4)	0.7482 (19)	0.074 (6)*	
O3	0.39039 (6)	0.63708 (15)	1.01569 (7)	0.02562 (19)	
H3C	0.4235 (13)	0.685 (3)	0.9772 (17)	0.062 (6)*	
H3D	0.3929 (13)	0.744 (4)	1.0688 (17)	0.062 (6)*	
O2	0.50431 (6)	0.71399 (15)	0.88618 (7)	0.02490 (19)	
H2C	0.5355 (14)	0.830 (4)	0.8673 (17)	0.066 (6)*	
H2D	0.5419 (14)	0.609 (4)	0.9175 (17)	0.063 (6)*	
N1	0.71223 (6)	0.77354 (16)	0.76435 (7)	0.01927 (19)	
C1	0.65506 (8)	0.5945 (2)	0.70453 (10)	0.0274 (2)	
H1A	0.616934	0.656109	0.635647	0.041*	
H1B	0.620479	0.536110	0.751545	0.041*	
H1C	0.689597	0.471912	0.686470	0.041*	
C2	0.77022 (7)	0.68697 (19)	0.86859 (9)	0.0219 (2)	
H2A	0.811282	0.578162	0.851550	0.026*	
H2B	0.736418	0.607142	0.911910	0.026*	
C3	0.81920 (7)	0.8817 (2)	0.93665 (8)	0.0219 (2)	
H3A	0.779377	0.970548	0.967625	0.026*	
H3B	0.864966	0.820396	0.999865	0.026*	
C4	0.85940 (6)	1.03588 (18)	0.86857 (8)	0.0176 (2)	
C5	0.82967 (6)	1.03240 (18)	0.75240 (8)	0.0173 (2)	
C6	0.76234 (7)	0.86499 (19)	0.69280 (8)	0.0201 (2)	
H6A	0.7237 (9)	0.935 (2)	0.6266 (12)	0.025 (3)*	
H6B	0.7924 (9)	0.735 (3)	0.6644 (12)	0.031 (4)*	
C7	0.92500 (7)	1.18598 (19)	0.91984 (8)	0.0198 (2)	
H7	0.945826	1.188163	0.998746	0.024*	
C8	0.96030 (7)	1.33188 (19)	0.85767 (9)	0.0213 (2)	
H8	1.005049	1.432594	0.893817	0.026*	
C9	0.92978 (7)	1.33005 (19)	0.74177 (9)	0.0220 (2)	
H9	0.953393	1.429876	0.698493	0.026*	
C10	0.86485 (7)	1.18171 (19)	0.69038 (8)	0.0204 (2)	
H10	0.843808	1.181363	0.611478	0.024*	

### Atomic displacement parameters (Å^2^)

**Table:**

	*U*^11^	*U*^22^	*U*^33^	*U*^12^	*U*^13^	*U*^23^
O1	0.0237 (4)	0.0211 (4)	0.0236 (4)	0.0030 (3)	0.0079 (3)	−0.0008 (3)
O3	0.0303 (4)	0.0251 (4)	0.0223 (4)	−0.0020 (3)	0.0086 (3)	−0.0023 (3)
O2	0.0270 (4)	0.0234 (4)	0.0255 (4)	−0.0006 (3)	0.0091 (3)	0.0030 (3)
N1	0.0197 (4)	0.0194 (4)	0.0192 (4)	−0.0010 (3)	0.0063 (3)	−0.0001 (3)
C1	0.0287 (6)	0.0240 (6)	0.0301 (6)	−0.0063 (5)	0.0092 (5)	−0.0064 (4)
C2	0.0236 (5)	0.0219 (5)	0.0219 (5)	0.0026 (4)	0.0091 (4)	0.0053 (4)
C3	0.0212 (5)	0.0287 (6)	0.0163 (4)	0.0002 (4)	0.0063 (4)	0.0056 (4)
C4	0.0176 (5)	0.0208 (5)	0.0158 (4)	0.0038 (4)	0.0068 (4)	0.0026 (4)
C5	0.0172 (5)	0.0199 (5)	0.0160 (4)	0.0038 (4)	0.0066 (4)	0.0015 (4)
C6	0.0216 (5)	0.0237 (5)	0.0157 (4)	−0.0008 (4)	0.0065 (4)	−0.0004 (4)
C7	0.0197 (5)	0.0243 (5)	0.0160 (4)	0.0034 (4)	0.0059 (4)	0.0001 (4)
C8	0.0199 (5)	0.0221 (5)	0.0230 (5)	−0.0010 (4)	0.0077 (4)	−0.0024 (4)
C9	0.0247 (5)	0.0230 (5)	0.0217 (5)	0.0008 (4)	0.0122 (4)	0.0031 (4)
C10	0.0231 (5)	0.0241 (5)	0.0157 (4)	0.0023 (4)	0.0084 (4)	0.0021 (4)

### Geometric parameters (Å, º)

**Table:**

O1—H1D	0.94 (2)	C3—H3B	0.9900
O1—H1E	0.95 (2)	C3—C4	1.5124 (14)
O3—H3C	0.86 (2)	C4—C5	1.3977 (13)
O3—H3D	0.91 (2)	C4—C7	1.3964 (15)
O2—H2C	0.92 (2)	C5—C6	1.5081 (15)
O2—H2D	0.88 (2)	C5—C10	1.3968 (14)
N1—C1	1.4678 (14)	C6—H6A	0.980 (14)
N1—C2	1.4708 (14)	C6—H6B	1.022 (15)
N1—C6	1.4671 (13)	C7—H7	0.9500
C1—H1A	0.9800	C7—C8	1.3865 (15)
C1—H1B	0.9800	C8—H8	0.9500
C1—H1C	0.9800	C8—C9	1.3952 (15)
C2—H2A	0.9900	C9—H9	0.9500
C2—H2B	0.9900	C9—C10	1.3833 (16)
C2—C3	1.5190 (16)	C10—H10	0.9500
C3—H3A	0.9900		
			
H1D—O1—H1E	106.2 (17)	C4—C3—H3B	109.1
H3C—O3—H3D	105.7 (18)	C5—C4—C3	119.87 (10)
H2C—O2—H2D	106.2 (18)	C7—C4—C3	121.11 (9)
C1—N1—C2	110.91 (9)	C7—C4—C5	119.01 (9)
C6—N1—C1	109.43 (8)	C4—C5—C6	121.09 (9)
C6—N1—C2	110.12 (8)	C10—C5—C4	119.45 (10)
N1—C1—H1A	109.5	C10—C5—C6	119.44 (9)
N1—C1—H1B	109.5	N1—C6—H6A	109.8 (8)
N1—C1—H1C	109.5	N1—C6—H6B	109.3 (8)
H1A—C1—H1B	109.5	C5—C6—H6A	110.2 (8)
H1A—C1—H1C	109.5	C5—C6—H6B	108.5 (8)
H1B—C1—H1C	109.5	H6A—C6—H6B	106.1 (11)
N1—C2—H2A	109.7	C4—C7—H7	119.4
N1—C2—H2B	109.7	C8—C7—C4	121.21 (9)
N1—C2—C3	109.72 (9)	C8—C7—H7	119.4
H2A—C2—H2B	108.2	C7—C8—H8	120.2
C3—C2—H2A	109.7	C7—C8—C9	119.67 (10)
C3—C2—H2B	109.7	C9—C8—H8	120.2
C2—C3—H3A	109.1	C8—C9—H9	120.3
C2—C3—H3B	109.1	C10—C9—C8	119.46 (10)
H3A—C3—H3B	107.8	C10—C9—H9	120.3
N1—C6—C5	112.77 (8)	C5—C10—H10	119.4
C4—C3—C2	112.45 (9)	C9—C10—C5	121.19 (10)
C4—C3—H3A	109.1	C9—C10—H10	119.4
			
N1—C2—C3—C4	−49.12 (12)	C4—C5—C10—C9	1.27 (16)
C1—N1—C2—C3	−171.72 (9)	C4—C7—C8—C9	0.23 (16)
C1—N1—C6—C5	−173.75 (9)	C5—C4—C7—C8	0.56 (16)
C2—N1—C6—C5	−51.59 (12)	C6—N1—C2—C3	67.00 (11)
C2—C3—C4—C5	18.69 (14)	C6—C5—C10—C9	−176.74 (10)
C2—C3—C4—C7	−162.56 (10)	C7—C4—C5—C6	176.68 (9)
C3—C4—C5—C6	−4.55 (15)	C7—C4—C5—C10	−1.29 (15)
C3—C4—C5—C10	177.48 (10)	C7—C8—C9—C10	−0.28 (16)
C3—C4—C7—C8	−178.19 (10)	C8—C9—C10—C5	−0.47 (16)
C4—C5—C6—N1	20.70 (14)	C10—C5—C6—N1	−161.33 (9)

### Hydrogen-bond geometry (Å, º)

**Table:**

*D*—H···*A*	*D*—H	H···*A*	*D*···*A*	*D*—H···*A*
O1—H1*D*···N1	0.94 (2)	1.81 (2)	2.7394 (12)	170.7 (18)
O1—H1*E*···O2^i^	0.95 (2)	1.81 (2)	2.7635 (12)	176 (2)
O3—H3*C*···O2	0.86 (2)	1.96 (2)	2.8070 (13)	165.8 (19)
O3—H3*D*···O1^ii^	0.91 (2)	1.84 (2)	2.7461 (12)	175.1 (19)
O2—H2*C*···O1	0.92 (2)	1.84 (2)	2.7538 (12)	174.9 (19)
O2—H2*D*···O3^iii^	0.88 (2)	1.88 (2)	2.7512 (13)	173 (2)
C6—H6*A*···O3^i^	0.98 (2)	2.49 (2)	3.4531 (3)	168.8 (2)

Symmetry codes: (i) −*x*+1, *y*+1/2, −*z*+3/2; (ii) −*x*+1, −*y*+2, −*z*+2; (iii) −*x*+1, −*y*+1, −*z*+2.

### Selected geometric parameter (Å,°)

**Table:**

N1–C1	1.4678 (14)	N1–C6	1.4671 (13)
N1–C2	1.4708 (14)	C4–C5	1.3977 (13)
			
C1–N1–C2	110.91 (9)	N1–C2–C3	109.72 (9)
C1–N1–C6	109.43 (8)	N1–C6–C5	112.77 (8)
C6–N1–C2	110.12 (8)	C4–C3–C2	112.45 (9)
